# Continuous vs. intermittent meropenem infusion in critically ill patients with sepsis: a systematic review and meta-analysis of randomized controlled trials with trial sequential analysis

**DOI:** 10.3389/fmed.2025.1580116

**Published:** 2025-06-10

**Authors:** Youquan Wang, Yanhua Li, Meng Gao, Mingtao Zhang, Yuting Li, Dongxia Wang, Xinyu Li, Chaoyang Zhang, Yao Fu, Hongxiang Li, Dong Zhang

**Affiliations:** ^1^Department of Critical Care Medicine, The First Hospital of Jilin University, Changchun, China; ^2^Department of Emergency Medicine, The Fifth Affiliated Hospital, Sun Yat-sen University, Zhuhai, China

**Keywords:** meropenem, continuous infusion, intermittent infusion, sepsis, critical illness

## Abstract

**Background:**

A recent large multicenter randomized controlled trial (RCT) found that continuous infusion (CI) of meropenem did not improve clinical outcomes in critically ill patients, contradicting previous meta-analysis results.

**Methods:**

We conducted a search of the PubMed, EMBASE, and Cochrane databases up to March 19, 2024.

**Results:**

Our study included a total of 1,075 critically ill patients with sepsis from five RCTs. The primary outcome indicated that CI of meropenem did not reduce all-cause mortality in patients (RR = 0.89; 95% CI, 0.75–1.04; *P* = 0.15; Chi^2^= 5.75; *I*^2^ = 30%). The secondary outcomes revealed that compared to II of meropenem, patients receiving CI had shorter ICU length of stay (MD = –2.39; 95% CI, –2.98 to –1.81; *P* < 0.00001; Chi^2^= 6.63; *I*^2^ = 40%), higher clinical cure rates (RR = 1.88; 95% CI, 1.23–2.87; *P* = 0.004; Chi^2^ = 1.87; *I*^2^ = 0%), and shorter duration of meropenem therapy (MD = –0.86; 95% CI, –1.36 to –0.36; *P* = 0.0008; Chi^2^ = 3.65; *I*^2^ = 45%).

**Conclusion:**

In critically ill patients with sepsis, CI of meropenem did not reduce mortality but was associated with shorter ICU length of stays, higher clinical cure rates, and shorter duration of meropenem therapy. Further large-scale RCTs are needed to validate these findings.

**Systematic review registration:**

https://www.crd.york.ac.uk/PROSPERO/view/CRD42024528380, identifier CRD42024528380.

## 1 Background

Sepsis is a severe inflammatory syndrome characterized by a dysregulated host response to infection (1–3), serving as a significant contributor to both intensive care unit (ICU) admission and mortality among patients. Beta-lactam antibiotics represent the most commonly used class of antimicrobial agents in the current armamentarium against infectious diseases, constituting 65% of all injectable antibiotic prescriptions in the United States (4).

Beta-lactam antibiotics have a short half-life, and if administered over a brief period, the peak blood concentration typically rapidly decreases below the minimum inhibitory concentration. Prolonged exposure below the minimum inhibitory concentration may diminish therapeutic efficacy, potentially allowing residual bacterial populations to resume growth and facilitate the emergence of resistant strains (5). Therefore, guidelines recommend prolonging the administration time of beta-lactam antibiotics to extend the duration above the minimum inhibitory concentration and achieve enhanced antimicrobial effects (6, 7). The findings of multiple pharmacokinetic studies also support the notion of extending the administration time to improve the efficacy of beta-lactams (8, 9).

Recent meta-analyses have indicated that continuous infusion (CI) of beta-lactams can reduce mortality and improve clinical cure rates in septic patients (10–15). There was a recent BLING III RCT (*N* = 7031) did not find that continuous infusion of beta-lactams improved 90-day mortality in critically ill patients (16). In addition, a meta-analysis did not find a difference in mortality between the two types of infusion (17). However, these meta-analyses are subject to certain limitations: (1) Variations in the types of antibiotics used across the included studies may introduce some bias, as the diverse pharmacological characteristics, spectrum of activity, and pharmacokinetics of different classes of beta-lactam antibiotics could potentially impact clinical outcomes in patients. (2) The inclusion of retrospective cohort studies and observational studies in some of the meta-analyses may also lower the quality of evidence for the outcomes.(3) These meta-analyses did not incorporate trial sequential analysis (TSA), which could increase the potential for type I or type II errors, thereby reducing the credibility of the study conclusions. Moreover, TSA provides a means to calculate the necessary sample sizes for clinically significant results and offers valuable perspectives on the potential lack of benefit from future trials, thus informing the feasibility and selection of outcome measures (18–20).

Meropenem is one of the most commonly used types of beta-lactam antibiotics, with its post-antibiotic effect demonstrated across various pathogens (21). CI of meropenem has been shown to potentially increase bacterial clearance rates and improve clinical outcomes in septic patients (11, 22). However, a recent high-quality clinical study did not find any benefit in terms of clinical outcomes for patients receiving CI of meropenem (23). Although a recent meta-analysis did not find a difference in mortality between the two meropenem infusions (24), three of the studies included in this meta-analysis did not use meropenem as their primary antimicrobial agent, leading to this highly misleading conclusion (9, 25, 26).

Therefore, it is necessary to conduct this systematic review of RCTs with meta-analysis and TSA to compare the effects of CI and intermittent infusion (II) of meropenem in critically ill patients with sepsis. This will help clarify whether meropenem can improve the clinical outcomes of critically ill septic patients.

## 2 Methods

### 2.1 Protocol and registration

The present systematic review and meta-analysis was conducted in strict adherence to the Preferred Reporting Items for Systematic Reviews and Meta-Analyses (PRISMA) reporting guidelines (27) (Supplementary Table S1). The study protocol for this meta-analysis has been registered in PROSPERO (CRD42024528380) on April 6, 2024, and is publicly accessible.

### 2.2 Eligibility criteria

This review exclusively incorporated randomized controlled trials (RCTs) involving patients with severe sepsis who received meropenem antimicrobial therapy. The specific inclusion and exclusion criteria are detailed in Table 1.

**TABLE 1:** Criteria to choose studies for the review based on the population, intervention, comparator, outcomes and study designs (PICOS) structure.

	Inclusion criteria	Exclusion criteria
Population	Critically ill patients with sepsis or septic shock	Under 18 years of age
Intervention	Meropenem was administered by continuous infusion	Other β-lactam antibiotics were used as the main antibiotics
Comparator	Meropenem was administered by intermittent infusion	Other β-lactam antibiotics were used as the main antibiotics
Outcomes	Clinical outcomes^a^	Only pharmacokinetic outcome
Study design	Randomized controlled trial	Letters, reviews, comments, retrospective, crossover or observational study

ICU, Intensive care unit. ^a^Clinical outcomes such as mortality, ICU length of stay and clinical cure rate, among others.

### 2.3 Data sources and search strategies

A comprehensive literature search was conducted across three electronic databases (PubMed, EMBASE, and Cochrane Library) to identify original studies assessing the effectiveness and safety profile of continuous vs. intermittent meropenem administration in adult sepsis patients, with the search period extending from the inception of the databases through March 2024. Database-specific modifications were applied to the search strategy, which was developed in collaboration with our institutional librarians (detailed search strategy available in Supplementary Table S2). Following initial screening based on titles and abstracts, two independent investigators (YW and YL) performed full-text evaluations of potentially relevant studies. The research team additionally conducted a manual review of the reference lists in the included articles to identify further relevant studies. Any discrepancies between reviewers were adjudicated by a third researcher (HL). The study selection process was managed using EndNote 20.0 software.

### 2.4 Types of outcome measures

The primary outcome was all-cause mortality (ICU mortality, hospital mortality, 28 or 90-day mortality). When multiple mortality outcomes were available, we preferred ICU mortality, which is the most critical for critically ill patients. We gave preference to intention-to-treat (ITT) dataset in both ITT and clinically evaluable datasets, because the ITT dataset includes all patients who were randomly assigned to treatment and control groups, it reflects real-world treatment effects and is more representative and general.

The secondary outcomes were Length of ICU length of stay, clinical cure rate and duration of meropenem therapy. Weighted means were calculated based on the number of patients in each study.

### 2.5 Quality assessment

Two independent reviewers (YL and YW) evaluated the methodological quality of the included trials using the Cochrane risk of bias assessment tool. The assessment focused on the following domains to evaluate potential biases: (1) randomization sequence generation (selection bias), (2) allocation concealment (selection bias), (3) blinding of study personnel and participants (performance bias), (4) blinding of outcome assessors (performance bias), (5) completeness of data reporting, including avoidance of arbitrary patient exclusions and minimal loss to follow-up (attrition bias), (6) selective reporting bias, and (7) other potential sources of bias. Each domain was rated using a color-coded system: green for satisfactory performance, yellow for unclear performance, and red for unsatisfactory performance. Any discrepancies between the reviewers were resolved through consultation with a third reviewer (HL). The results of the risk of bias assessment are summarized in a graph and table, which are provided in Supplementary Figure S1.

### 2.6 Statistical analysis

Studies meeting the inclusion criteria and not violating the exclusion criteria were imported into Review Manager Version 5.3 (RevMan, The Cochrane Collaboration, Oxford, UK) for meta-analysis. For dichotomous outcomes, relative risk (RR) with 95% confidence intervals (CI) was calculated. For continuous outcomes, the mean difference (MD) with 95% CI was calculated as the effect measure. In cases where significant heterogeneity was detected, as indicated by a chi-squared test (*P* < 0.10) and an inconsistency index (*I*^2^ ≥ 50%) (28), a random-effects model was employed to pool the data. For continuous variables reported as median (interquartile range), the mean and standard deviation were estimated using a validated calculator (29) based on sample size, enabling their inclusion in the meta-analysis. Sensitivity analysis was conducted using Stata 16 to assess the stability and reliability of the results. Publication bias was evaluated through funnel plots and Egger’s weighted regression tests, with a *P*-value of < 0.05 considered statistically significant for determining the overall intervention effect.

### 2.7 Trial sequential analysis

In our meta-analysis, we utilized TSA to manage random errors and determine the conclusiveness of our findings. Employing a random effects model, we plotted the cumulative *Z* curve. The TSA was conducted to uphold a 5% risk of type I error overall. Based on previous high-quality RCTs in the field (22, 23), we assumed a 15.0% anticipated relative risk reduction (RRR) with 90% power to calculate the necessary information size for detecting or refuting an intervention effect. By adjusting the control event rate based on the relevant data from the II group in our meta-analysis, we monitored the cumulative *Z* curve’s trajectory. If the curve intersected the trial sequential monitoring boundary or entered the futility zone, it suggested sufficient evidence to either accept or reject the expected intervention effect, eliminating the need for further studies. Conversely, if the Z curve did not breach any boundaries and the required information size had not been attained, the evidence would be deemed insufficient for drawing a conclusion, indicating the necessity for additional research (30).

## 3 Results

### 3.1 Study selection process

**FIGURE 1:**
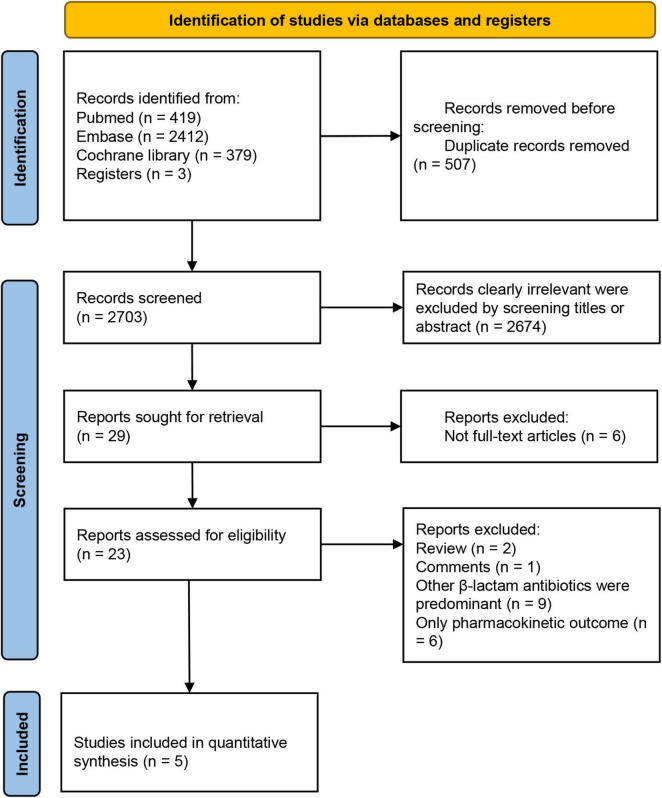
PRISMA flow chart on selection and inclusion of studies.

The initial search yielded 3,210 potential studies, with three additional studies identified through cross-referencing and the authors’ personal reference collections. Following the removal of 507 duplicates, 2,703 manuscripts were screened based on titles and abstracts, and 29 trials underwent full-text review. The study selection process is illustrated in Figure 1. The studies that met the inclusion criteria but not the exclusion criteria, and the reasons for exclusion in each study are presented in Supplementary Table S3. Five RCTs met the inclusion criteria for this review and were included for data extraction. These studies included one conducted in the Czech Republic, two in China, one in Egypt, and one conducted across Croatia, Italy, Kazakhstan, and Russia. Four of the studies were single-center trials, while one was a multicenter study. Detailed characteristics of the trials and their participants are provided in Table 2 and Supplementary Table S4, respectively (22, 23, 31–33). In addition, we counted the type of infection and the proportion of patients in these studies (Supplementary Table S5).

**TABLE 2:** Characteristics of the trials included in this review (*n* = 5).

Included trials and years	Country	Study design	Participants (*n*) CI vs. II	First day dose of meropenem (g) CI vs. II	Administration scheme of CI group	Administration scheme of II group	Primary outcomes
Chytra et al. (22) 2012	Czech Republic	Multicenter Open-label	120 vs. 120	6.0 vs. 6.0	2.0 g loading dose, 4.0 g continuous infusion for 24 h	2 g meropenem 0.5 h infusion, once every 8 h	Clinical cure was comparable between both groups. Microbiological success rate was higher in the CI group.
Wang et al.(32) 2014	China	Single-center Single blind	38 vs. 40	3.0 vs. 3.0	0.25 g loading dose for 10 min, 0.75 g continuous infusion for 3 h, once every 8 h	1 g meropenem 0.5 h infusion, once every 8 h	The CI group had higher clinical cure rate and lower 28-day mortality.
Mohamed et al. (31) 2015	Egypt	Single-center Blind: NR	50 vs. 50	6.0 vs. 6.0	2.0 g loading dose, 4.0 g continuous infusion for 24 h	2 g meropenem 0.5 h infusion, once every 8 h	The CI group associated with significant reduction of WBCs count, CRP levels, SOFA score and ICU length of stay.
Zhao et al.(33) 2017	China	Single-center Single blind	25 vs. 25	3.0 vs. 3.5	0.5 g loading dose, 3.0 g continuous infusion for 24 h	1.5 g loading dose followed by 1.0 g meropenem 0.5 h infusion, once every 8 h	There was no difference in clinical cure rate and microbial eradication rate between the two groups
Monti et al. (23) 2023	Croatia, Italy, Kazakhstan, and Russia	Multicenter Double-blind	303 vs. 304	4.0 vs. 4.0	A loading dose of 1 g of meropenem followed by 3 g over 24 h	A loading dose of 1 g of meropenem followed by 1 g every 8 h (infusion over 30–60 min)	The continuous administration of meropenem did not improve the composite outcome of mortality and emergence of pandrug-resistant or extensively drug-resistant bacteria at day 28.

CI, Continuous infusion; II, Intermittent infusion; NR, not reported.

### 3.2 Primary outcome

**FIGURE 2:**
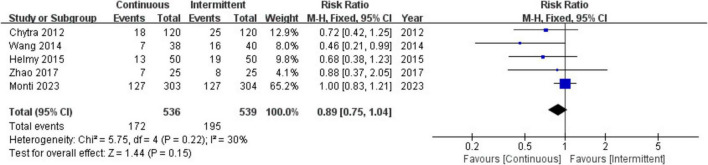
Forest plot for all-cause mortality.

Five trials involving 1,075 patients reported all-cause mortality in both the continuous infusion (CI) and intermittent infusion (II) groups and were included in the meta-analysis. The results indicated no significant difference in all-cause mortality between the CI and II groups (RR = 0.89; 95% CI, 0.75–1.04; *P* = 0.15; Chi^2^ = 5.75; *I*^2^ = 30%) (Figure 2).

### 3.3 Secondary outcomes

#### 3.3.1 ICU length of stay

**FIGURE 3:**
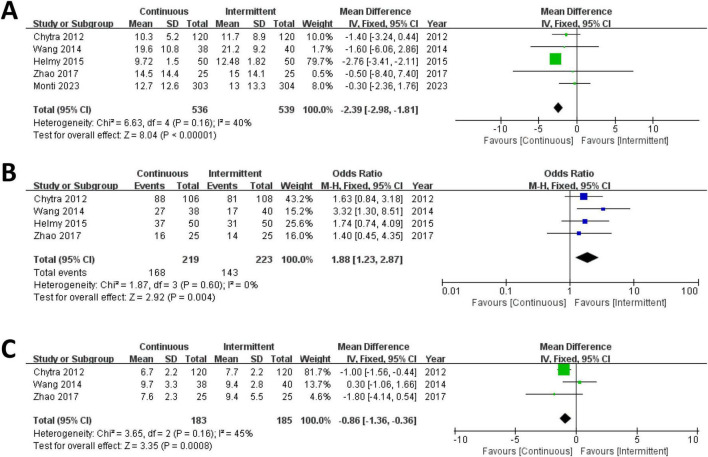
Forest plot for ICU length of stay **(A)**, clinical cure rate **(B)** and duration of meropenem therapy **(C)**.

Five trials involving 1,075 patients reported ICU length of stay for both the CI and II groups and were included in the meta-analysis. The results demonstrated that the ICU length of stay was significantly shorter in the CI group compared to the II group (MD = –2.39; 95% CI, –2.98 to –1.81; *P* < 0.00001; Chi^2^ = 6.63; *I*^2^ = 40%) (Figure 3A).

#### 3.3.2 Clinical cure rate

Four trials involving 442 patients reported clinical cure rates for both the CI and II groups and were included in the meta-analysis. The results indicated that the clinical cure rate was significantly higher in the CI group compared to the II group (RR = 1.88; 95% CI, 1.23–2.87; *P* = 0.004; Chi^2^ = 1.87; *I*^2^ = 0%) (Figure 3B).

#### 3.3.3 Duration of meropenem therapy

Three trials involving 368 patients reported the duration of meropenem therapy for both the CI and II groups and were included in the meta-analysis. The results revealed that the duration of meropenem therapy was significantly shorter in the CI group compared to the II group (MD = –0.86; 95% CI, –1.36 to –0.36; *P* = 0.0008; Chi^2^ = 3.65; *I*^2^ = 45%) (Figure 3C).

### 3.4 Risk of bias and sensitivity analyses within outcomes

Funnel plots were utilized to evaluate publication bias across all outcomes, with no significant bias detected for any outcome. This conclusion was supported by the funnel plot results (*I*^2^ < 50%) and Egger’s test (*P* > 0.05) (Supplementary Figure S2). To assess the robustness of the findings, a sensitivity analysis was conducted by sequentially excluding one study at a time and recalculating the combined effect sizes for the remaining studies. The direction and magnitude of the pooled estimates remained consistent regardless of the exclusion of any single study. Sensitivity analyses confirmed the stability of the results for both the primary and secondary outcomes (Supplementary Figure S3).

### 3.5 Trial sequential analysis

**FIGURE 4:**
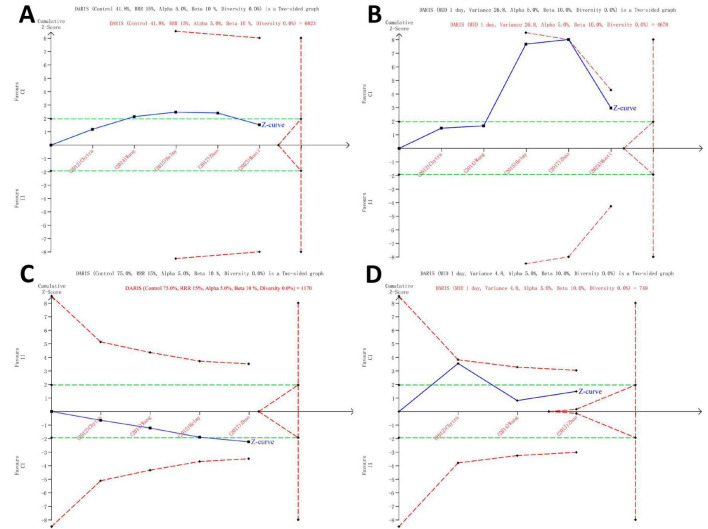
Trial Sequential Analysis of Clinical Outcomes. All-cause mortality in all patients (5 studies, *n* = 1075) **(A)**, ICU length of stay (5 studies, *n* = 1075) **(B)**, clinical cure rate (4 studies, *n* = 442) **(C)** and duration of meropenem therapy (3 studies, *n* = 368) **(D)**. TSA was analyzed using Der Simonian and Laird random-effects model. The *Z* curve in blue measures the treatment effect (pooled relative risk). The parallel lines in green are the boundaries of conventional meta-analysis (alpha 5%), and the boundaries of benefit and harm are boundaries of conventional meta-analysis adjusted for between-trial heterogeneity and multiple statistical testing (TSA boundaries). A treatment effect outside the TSA boundaries of benefit/harm indicates reliable evidence for a treatment effect, and a treatment effect within the futility zone (the triangle between the parallel lines) indicates that there is reliable evidence of no treatment effect. DARIS: diversity adjusted required information size is the calculated optimum sample size for statistical inference, MID: minimally important difference, RRR: relative risk reduction, TSA: trial sequential analysis.

The findings of the TSA are detailed in Supplementary Table S6 and illustrated in Figure 4, indicating that the current systematic review did not reach the necessary information sizes to identify the pre-specified effect sizes for all-cause mortality, ICU length of stay, clinical cure rate, and duration of meropenem therapy. In the TSA of all-cause mortality, a transient significance is observed in the *Z*-curve (Figure 4A), where the initially detected effect may appear significant but gradually diminishes and eventually disappears as the data volume increases or other factors come into play. Researchers should exercise caution in interpreting the results, taking into consideration the stability and persistence of the effect, indicating the potential necessity for further research to validate these outcomes.

## 4 Discussion

This systematic review and meta-analysis of 5 RCTs, including 1,075 patients, the effects of CI and II of meropenem on the clinical outcomes of critically ill patients with sepsis were compared. This meta-analysis focused on the primary outcome (all-cause mortality) in critically ill patients with sepsis. The study findings did not reveal a significant difference in all-cause mortality between the CI group and the II group. Interestingly, this result contradicts the meta-analysis results reported by Roberts et al. (12), Zhao et al. (10) and other meta-analyses (11, 13). Our study also found that the CI group had a shorter ICU length of stay, a higher clinical cure rate, and a shorter duration of meropenem therapy compared to the II group. The three secondary outcomes of this study, which share some similarities with previous meta-analytical findings. However, TSA indicated that more trials are needed to further confirm these findings.

Prior to this meta-analysis, there have been multiple meta-analyses specifically comparing the clinical outcomes of septic patients CI vs. II of beta-lactam antibiotics or meropenem (10–14). Although they had differences in their inclusion and exclusion criteria, they all arrived at a consistent clinical outcome: compared to intermittent infusion, CI beta-lactam antibiotics or meropenem can reduce mortality in septic patients. The different choices of beta-lactam antibiotics may indicate the heterogeneity of the condition, and focusing on the use of a single antibiotic may reduce the impact of this heterogeneity. Currently, there is a lack of systematic review and meta-analysis of RCTs comparing CI vs. II of meropenem in critically ill patients with sepsis. Therefore, we sought to address this gap through our meta-analysis. Interestingly, we arrived at an unexpected conclusion: CI of meropenem did not reduce mortality in critically ill septic patients. This finding is consistent with the results of a recent high-quality RCTs (23), as well as a study by Dulhunty et al. (25), which included 25 ICUs and 432 critically ill patients and found that CI or II beta-lactam antibiotics had no impact on mortality in critically ill patients. Several similar RCTs also failed to find a significant difference in mortality between the two infusion methods (22, 34, 35). While the meta-analysis conducted by Falagas et al. (14) indicated that CI beta-lactam antibiotics can reduce mortality in septic patients, it should be noted that this analysis included some non-severe cases. The pharmacokinetic and pharmacodynamic advantages of CI of meropenem are indisputable. Moreover, prolonged or continuous administration of meropenem, with higher plasma and tissue concentrations, particularly against resistant pathogens such as *Acinetobacter* spp. and *Pseudomonas aeruginosa*, can provide greater exposure to achieve optimal antimicrobial efficacy (8, 36, 37). However, the management of severe sepsis is a complex endeavor: factors such as the source and type of infection, the severity of the patient’s illness, and even differences in diagnostic and treatment practices all play crucial roles in determining clinical outcomes. Therefore, based on the results of our study’s meta-analysis of RCTs, the conclusion that CI of meropenem can reduce mortality in patients cannot be definitively drawn. In addition, TSA indicated that there may be a type-II error, and the effect of the two infusion modes on mortality in patients with severe sepsis needs to be validated by further studies.

CI of meropenem, at equivalent dosages, can enhance its antimicrobial efficacy. The secondary outcomes of this study indicate a higher clinical cure rate in the CI group, consistent with previous meta-analysis outcomes (11, 13). The improved antimicrobial efficacy expedites the antimicrobial treatment process, leading to a shorter duration of meropenem therapy in the CI group, in line with the results reported by Chytra et al. (22). Additionally, the meta-analysis results revealed a shorter ICU stay in the CI group, akin to the findings of Nicasio et al. (38) and other studies (31, 39), despite the absence of this conclusion in larger RCTs (23, 25, 40). ICU length of stay and mortality, being indirect clinical outcomes influenced by meropenem antimicrobial therapy for infections, may be affected by multiple covariates. Therefore, the variability of these outcomes across different studies is understandable. Although there is minimal heterogeneity (*I*^2^ < 50% and Egger’s test *P* > 0.05) between the studies reporting these secondary outcomes, caution is warranted in interpreting the results due to the limited number of studies and patients included, as well as a type-1 error demonstrated by TSA in all secondary outcomes. Further high-quality RCTs are necessary to validate these two conclusions.

### Strengths and limitations

This study has several strengths: (1) Different from other meta-analyses, we exclusively included studies utilizing meropenem as the primary antimicrobial agent, significantly reducing the influence of different antibiotics on the clinical outcomes in the meta-analysis; (2) This study only included RCTs, excluding low-quality studies such as retrospective and observational studies; (3) We incorporated the latest RCT with a total sample size of 607 patients, representing the highest quality study to date (23); (4) The use of TSA enabled us to identify the risk of type-I or type-II errors in our findings. The diversity adjusted required information size (DARIS) estimated from TSA will also inform the sample size needed for adequately powered future trials. These strengths will enhance the stability and value of the conclusions.

This study also has several limitations: (1) Although we constructed funnel plots to assess heterogeneity and both the *I*^2^ value and Egger’s test supported minimal heterogeneity among the studies, it is noteworthy that out of the 5 included RCTs, 3 were single-center studies and not all studies employed a double-blind experimental design, which may introduce bias to the conclusions; (2) For the primary outcome of all-cause mortality rate, despite establishing prioritized selection, we combined different mortality rate outcomes, inevitably introducing bias; (3) While all included studies primarily utilized meropenem as the main antimicrobial agent, there were variations in the distribution of infection types, disease severity (APACHE II, SOFA, and SAPS II scores), and other baseline data among the studies, which are important factors influencing clinical outcomes; (4) Despite conducting a comprehensive search for studies utilizing meropenem as the main antimicrobial agent, only 5 RCTs were included in this study, with a relatively small overall sample size. Additionally, some studies had sample sizes of only a few dozen cases, further contributing to outcome uncertainty; (5) Due to the small number of included studies and sample size, and because all studies were not focused on a specific type of infection, no subgroup analysis was conducted. However, the impact of factors such as different infection types, sites, and disease categories on outcomes cannot be ignored. This limitation should be addressed in future high-quality studies. In summary, cautious interpretation of the results of this study is warranted, and further research is needed to validate the conclusions drawn.

Given the current research and its limitations, we attempt to propose some future directions: (1) Conduct larger, multicenter, double-blind RCTs with standardized protocols for mortality definitions, PK/PD monitoring, and subgroup analyses. (2) Consider economic effects to quantify cost-effectiveness and clinical outcomes. (3) CI theoretically offers advantages; it is necessary to explore patient populations (subgroup analyses) that would benefit from CI, rather than continuing clinical studies targeting all patient groups. The importance of personalized treatment should be emphasized.

## 5 Conclusion

In critically ill patients with sepsis, compared to II administration, CI of meropenem did not reduce mortality. However, CI of meropenem was associated with a reduction in ICU length of stay, an increase in clinical cure rates, and a shorter duration of meropenem therapy. Further large-scale randomized controlled trials evaluating the effects of CI and II on clinical outcomes in critically ill patients with sepsis are needed to confirm these results.

## Data Availability

Publicly available datasets were analyzed in this study. This data can be found here: not applicable.
