# Supplementary material for: Continuous vs. intermittent meropenem infusion in critically ill patients with sepsis: a systematic review and meta-analysis of randomized controlled trials with trial sequential analysis
Source: Front Med (Lausanne). 2025 Jun 10;12:1580116. doi: 10.3389/fmed.2025.1580116 (PMC12185501; doi:10.3389/fmed.2025.1580116)
Supplement: Supplementary file 1 [file Table_1.docx]

**Supplemental Information**

Supplemental Table 1: PRISMA Checklist.

Supplemental Table 2: Search Strategy for Each Database.

Supplemental Table 3: Studies Meeting Inclusion Criteria but Excluded: Reasons for Exclusion.

Supplemental Table 4: Characteristics of the Included Participants in each trial.

Supplemental Table 5: Type of infection and the proportion of patients.

Supplemental Table 6: Summary of results of trial sequential analyses.

Supplemental Figure 1. Risk of bias graph and risk of bias summary graph.

Supplemental Figure 2. Funnel plots.

Supplemental Figure 3. Sensitivity analyses clinical and nutritional outcomes.

Supplemental Table 1: PRISMA Checklist.

| **Section/topic** | **#** | **Checklist item** | **Reported on page #** |
| --- | --- | --- | --- |
| **TITLE** | | |  |
| Title | 1 | Identify the report as a systematic review, meta-analysis, or both. | 1 |
| **ABSTRACT** | | |  |
| Structured summary | 2 | Provide a structured summary including, as applicable: background; objectives; data sources; study eligibility criteria, participants, and interventions; study appraisal and synthesis methods; results; limitations; conclusions and implications of key findings; systematic review registration number. | 3-4 |
| **INTRODUCTION** | | |  |
| Rationale | 3 | Describe the rationale for the review in the context of what is already known. | 5-6 |
| Objectives | 4 | Provide an explicit statement of questions being addressed with reference to participants, interventions, comparisons, outcomes, and study design (PICOS). | 5-6 |
| **METHODS** | | |  |
| Protocol and registration | 5 | Indicate if a review protocol exists, if and where it can be accessed (e.g., Web address), and, if available, provide registration information including registration number. | 7 |
| Eligibility criteria | 6 | Specify study characteristics (e.g., PICOS, length of follow-up) and report characteristics (e.g., years considered, language, publication status) used as criteria for eligibility, giving rationale. | 7 |
| Information sources | 7 | Describe all information sources (e.g., databases with dates of coverage, contact with study authors to identify additional studies) in the search and date last searched. | 7-8 |
| Search | 8 | Present full electronic search strategy for at least one database, including any limits used, such that it could be repeated. | 8 |
| Study selection | 9 | State the process for selecting studies (i.e., screening, eligibility, included in systematic review, and, if applicable, included in the meta-analysis). | 8-9 |
| Data collection process | 10 | Describe method of data extraction from reports (e.g., piloted forms, independently, in duplicate) and any processes for obtaining and confirming data from investigators. | 8-9 |
| Data items | 11 | List and define all variables for which data were sought (e.g., PICOS, funding sources) and any assumptions and simplifications made. | 8-9 |
| Risk of bias in individual studies | 12 | Describe methods used for assessing risk of bias of individual studies (including specification of whether this was done at the study or outcome level), and how this information is to be used in any data synthesis. | 9 |
| Summary measures | 13 | State the principal summary measures (e.g., risk ratio, difference in means). | 10 |
| Synthesis of results | 14 | Describe the methods of handling data and combining results of studies, if done, including measures of consistency (e.g., I^2^) for each meta-analysis. | 10 |
| Risk of bias across studies | 15 | Specify any assessment of risk of bias that may affect the cumulative evidence (e.g., publication bias, selective reporting within studies). | 9 |
| Additional analyses | 16 | Describe methods of additional analyses (e.g., sensitivity or subgroup analyses, meta-regression), if done, indicating which were pre-specified. | 10-11 |
| **RESULTS** | | |  |
| Study selection | 17 | Give numbers of studies screened, assessed for eligibility, and included in the review, with reasons for exclusions at each stage, ideally with a flow diagram. | 11-12 |
| Study characteristics | 18 | For each study, present characteristics for which data were extracted (e.g., study size, PICOS, follow-up period) and provide the citations. | 13 |
| Risk of bias within studies | 19 | Present data on risk of bias of each study and, if available, any outcome level assessment (see item 12). | 9 |
| Results of individual studies | 20 | For all outcomes considered (benefits or harms), present, for each study: (a) simple summary data for each intervention group (b) effect estimates and confidence intervals, ideally with a forest plot. | 14-19 |
| Synthesis of results | 21 | Present results of each meta-analysis done, including confidence intervals and measures of consistency. | 14-19 |
| Risk of bias across studies | 22 | Present results of any assessment of risk of bias across studies (see Item 15). | 16 |
| Additional analysis | 23 | Give results of additional analyses, if done (e.g., sensitivity or subgroup analyses, meta-regression [see Item 16]). | 16-19 |
| **DISCUSSION** | | |  |
| Summary of evidence | 24 | Summarize the main findings including the strength of evidence for each main outcome; consider their relevance to key groups (e.g., healthcare providers, users, and policy makers). | 20-22 |
| Limitations | 25 | Discuss limitations at study and outcome level (e.g., risk of bias), and at review-level (e.g., incomplete retrieval of identified research, reporting bias). | 22-23 |
| Conclusions | 26 | Provide a general interpretation of the results in the context of other evidence, and implications for future research. | 23 |
| **FUNDING** | | |  |
| Funding | 27 | Describe sources of funding for the systematic review and other support (e.g., supply of data); role of funders for the systematic review. | 35 |

*From:* Moher D, Liberati A, Tetzlaff J, Altman DG, The PRISMA Group (2009). Preferred Reporting Items for Systematic Reviews and Meta-Analyses: The PRISMA Statement. PLoS Med 6(6): e1000097. doi:10.1371/journal.pmed1000097

Supplemental Table 2. Search Strategy for Each Database

| Database | Search strategy | Results |
| --- | --- | --- |
| Pubmed | #1 ((((((((((((((((((((((((((((((("Meropenem"[MeSH Terms] OR "beta-Lactams"[MeSH Terms] OR "Carbapenems"[MeSH Terms] OR "Cephalosporins"[MeSH Terms] OR "Clavulanic Acids"[MeSH Terms] OR "Monobactams"[MeSH Terms] OR "Moxalactam"[MeSH Terms] OR "Penicillins"[MeSH Terms]) OR (beta Lactams[Title/Abstract])) OR (beta-Lactam[Title/Abstract])) OR (beta Lactam[Title/Abstract])) OR (Antibiotics, Carbapenem[Title/Abstract])) OR (Carbapenem Antibiotics[Title/Abstract])) OR (Carbapenem[Title/Abstract])) OR (Antibiotics, Cephalosporin[Title/Abstract])) OR (Cephalosporin Antibiotics[Title/Abstract])) OR (Cephalosporin Antibiotic[Title/Abstract])) OR (Antibiotic, Cephalosporin[Title/Abstract])) OR (Cephalosporin[Title/Abstract])) OR (Cephalosporanic Acids[Title/Abstract])) OR (Acids, Cephalosporanic[Title/Abstract])) OR (Cephalosporanic Acid[Title/Abstract])) OR (Acid, Cephalosporanic[Title/Abstract])) OR (Acids, Clavulanic[Title/Abstract])) OR (Antibiotics, Monobactam[Title/Abstract])) OR (Monobactam Antibiotics[Title/Abstract])) OR (Monocyclic beta-Lactams[Title/Abstract])) OR (Monocyclic beta Lactams[Title/Abstract])) OR (Monocyclic beta-Lactam[Title/Abstract])) OR (Monocyclic beta Lactam[Title/Abstract])) OR (beta-Lactam, Monocyclic[Title/Abstract])) OR (Monobactam[Title/Abstract])) OR (beta-Lactams, Monocyclic[Title/Abstract])) OR (beta Lactams, Monocyclic[Title/Abstract])) OR (Lamoxactam[Title/Abstract])) OR (Latamoxef[Title/Abstract])) OR (Antibiotics, Penicillin[Title/Abstract])) OR (Penicillin Antibiotics[Title/Abstract])) OR (Penicillin[Title/Abstract]) | [185,510](https://pubmed.ncbi.nlm.nih.gov/?term=longqueryd83bbfec4b643d4a4e86&sort=) |
|  | #2 (((((((continuous[Title/Abstract]) OR (extended[Title/Abstract])) OR (prolonged[Title/Abstract])) OR (intermittent[Title/Abstract])) OR (interval[Title/Abstract])) OR (discontinuous[Title/Abstract])) OR (bolus[Title/Abstract])) OR (pulse[Title/Abstract]) | [2,165,967](https://pubmed.ncbi.nlm.nih.gov/?term=%28%28%28%28%28%28%28continuous%5BTitle%2FAbstract%5D%29+OR+%28extended%5BTitle%2FAbstract%5D%29%29+OR+%28prolonged%5BTitle%2FAbstract%5D%29%29+OR+%28intermittent%5BTitle%2FAbstract%5D%29%29+OR+%28interval%5BTitle%2FAbstract%5D%29%29+OR+%28discontinuous%5BTitle%2FAbstract%5D%29%29+OR+%28bolus%5BTitle%2FAbstract%5D%29%29+OR+%28pulse%5BTitle%2FAbstract%5D%29&sort=) |
|  | #3 ((((((((((((((((((((((((((((("Sepsis"[Mesh]) OR "Shock, Septic"[Mesh]) OR (Bloodstream Infection[Title/Abstract])) OR (Bloodstream Infections[Title/Abstract])) OR (Infection, Bloodstream[Title/Abstract])) OR (Pyemia[Title/Abstract])) OR (Pyemias[Title/Abstract])) OR (Pyohemia[Title/Abstract])) OR (Pyohemias[Title/Abstract])) OR (Pyaemia[Title/Abstract])) OR (Pyaemias[Title/Abstract])) OR (Septicemia[Title/Abstract])) OR (Septicemias[Title/Abstract])) OR (Poisoning, Blood[Title/Abstract])) OR (Blood Poisoning[Title/Abstract])) OR (Blood Poisonings[Title/Abstract])) OR (Poisonings, Blood[Title/Abstract])) OR (Severe Sepsis[Title/Abstract])) OR (Sepsis, Severe[Title/Abstract])) OR (Septic Shock[Title/Abstract])) OR (Shock, Toxic[Title/Abstract])) OR (Toxic Shock Syndrome[Title/Abstract])) OR (Shock Syndrome, Toxic[Title/Abstract])) OR (Toxic Shock Syndromes[Title/Abstract])) OR (Toxic Shock[Title/Abstract])) OR (Shock, Endotoxic[Title/Abstract])) OR (Endotoxin Shock[Title/Abstract])) OR (Endotoxin Shocks[Title/Abstract])) OR (Shock, Endotoxin[Title/Abstract])) OR (Shocks, Endotoxin[Title/Abstract]) | 207,818 |
|  | #4 (((((((((("Critical Care"[Mesh]) OR (Care, Critical[Title/Abstract])) OR (Intensive Care[Title/Abstract])) OR (Care, Intensive[Title/Abstract])) OR (Surgical Intensive Care[Title/Abstract])) OR (Care, Surgical Intensive[Title/Abstract])) OR (Intensive Care, Surgical[Title/Abstract]))) OR ((((("Critical Illness"[Mesh]) OR (Critical Illnesses[Title/Abstract])) OR (Illness, Critical[Title/Abstract])) OR (Illnesses, Critical[Title/Abstract])) OR (Critically Ill[Title/Abstract]))) OR (((("Intensive Care Units"[Mesh]) OR (Intensive Care Unit[Title/Abstract])) OR (Unit, Intensive Care[Title/Abstract])) OR (ICU Intensive Care Units[Title/Abstract]))) | 312,834 |
|  | #5 #1 and #2 and #3 and #4 | [419](https://pubmed.ncbi.nlm.nih.gov/?term=longquery4fb6663d27d0e555ec3b&sort=) |
| Embase | #1 'beta lactam'/exp  #2 'carbapenem derivative'/exp  #3 'cephalosporin derivative'/exp  #4 'clavulanic acid'/exp  #5 'monobactam derivative'/exp  #6 'penicillin derivative'/exp  #7 'latamoxef'/exp  #8 ('2 [2beta':ab,ti AND dimethylaminocarbonyl:ab,ti AND 'pyrrolidin 4beta ylthio] 6alpha':ab,ti AND '1 hydroxyethyl':ab,ti AND '1beta methyl 1 dethia 1 carba 2 penem 3 carboxylic acid':ab,ti OR 'ici 194660':ab,ti OR 'mepem':ab,ti OR 'meronem':ab,ti OR 'meropen':ab,ti OR 'meropenem in 0.9% sodium chloride':ab,ti OR 'meropenem in sodium chloride 0.9%':ab,ti OR 'meropenem trihydrate':ab,ti OR 'merrem':ab,ti OR 'merrem i.v.':ab,ti OR 'sm 7338':ab,ti OR 'sm7338':ab,ti OR 'meropenem':ab,ti OR 'beta lactam ring':ab,ti OR 'beta lactams':ab,ti OR 'beta-lactams':ab,ti OR 'beta lactam':ab,ti OR 'antibiotics, carbapenems':ab,ti OR 'carbapenems':ab,ti OR 'carbapenem derivative':ab,ti OR '3` acetoxymethyl 3 cephem derivative':ab,ti OR 'antibiotics, cephalosporins':ab,ti OR 'cefalosporin derivative':ab,ti OR 'cephalosporin series':ab,ti OR 'cephalosporine derivative':ab,ti OR 'cephalosporins':ab,ti OR 'cephalosporin derivative':ab,ti OR 3:ab,ti) AND '2 hydroxyethylidene':ab,ti AND '7 oxo 4 oxa 1 azabicyclo [3.2.0] heptane 2 carboxylic acid':ab,ti OR 'brl 14151':ab,ti OR 'brl14151':ab,ti OR 'clavulanate':ab,ti OR 'clavulanic acids':ab,ti OR 'clavulanic acid':ab,ti OR 'antibiotics, monobactams':ab,ti OR 'monobactam':ab,ti OR 'monobactam antibiotic':ab,ti OR 'monobactams':ab,ti OR 'monobactam derivative':ab,ti OR 'antibiotics, penicillins':ab,ti OR 'penicillin':ab,ti OR 'penicillin derivate':ab,ti OR 'penicillin series':ab,ti OR 'penicillins':ab,ti OR 'penicillium extract':ab,ti OR 'penicillin derivative':ab,ti  #9 continuous:ab,ti OR extended:ab,ti OR prolonged:ab,ti OR intermittent:ab,ti OR interval:ab,ti OR discontinuous:ab,ti OR bolus:ab,ti OR pulse:ab,ti  #10 'sepsis'/exp  #11 'septic shock'/exp  #12 'abdominal sepsis':ab,ti OR 'focal sepsis':ab,ti OR 'intraabdominal sepsis':ab,ti OR 'sepsis syndrome':ab,ti OR 'septic disease':ab,ti OR 'sepsis':ab,ti OR 'sepsis-associated hypotension':ab,ti OR 'septicaemic shock':ab,ti OR 'septicemic shock':ab,ti OR 'shock, septic':ab,ti OR 'septic shock':ab,ti  #13 'intensive care'/exp  #14 'intensive care unit'/exp  #15 'critical illness'/exp  #16 'care, intensive':ab,ti OR 'critical care':ab,ti OR 'intensive care, paediatric':ab,ti OR 'intensive care, pediatric':ab,ti OR 'intensive therapy':ab,ti OR 'paediatric intensive care':ab,ti OR 'pediatric intensive care':ab,ti OR 'therapy, intensive':ab,ti OR 'intensive care':ab,ti OR 'close attention unit':ab,ti OR ('combined medical':ab,ti AND 'surgical icu':ab,ti) OR ('combined surgical':ab,ti AND 'medical icu':ab,ti) OR 'critical care unit':ab,ti OR 'general icu':ab,ti OR gicu:ab,ti OR gicus:ab,ti OR icus:ab,ti OR 'intensive care department':ab,ti OR 'intensive care units':ab,ti OR 'intensive therapy unit':ab,ti OR 'intensive treatment unit':ab,ti OR 'medical-surgery icu':ab,ti OR 'medical/surgical icu':ab,ti OR 'medical/surgical icus':ab,ti OR 'medico-surgical icu':ab,ti OR ('mixed medical':ab,ti AND 'surgical icu':ab,ti) OR ('mixed surgical':ab,ti AND 'medical icu':ab,ti) OR 'respiratory care unit':ab,ti OR 'respiratory care units':ab,ti OR 'special care unit':ab,ti OR 'surgery/medical icu':ab,ti OR 'surgical-medical icus':ab,ti OR 'surgical/medical icu':ab,ti OR 'unit, intensive care':ab,ti  #17 #1 or #2 or #3 or #4 or #5 or #6 or #7 or #8  #18 #10 or #11 or #12  #19 #13 or #14 or #15 or #16  #20 #17 and #9 and #18 or #19 | 10,237  12,197  293,300  16,418  22,279  409,756  5,938  108,296  2,903,014  362,735  75,366  221,527  939,107  311,170  36,401  352,257  575,202  407,830  1,215,262  2,412 |
| Cochrane  library | #1 MeSH descriptor: [Meropenem] explode all trees  #2 MeSH descriptor: [beta-Lactams] explode all trees  #3 MeSH descriptor: [beta Lactam Antibiotics] explode all trees  #4 MeSH descriptor: [Monobactams] explode all trees  #5 MeSH descriptor: [Cephalosporinase] explode all trees  #6 (“Antibiotics” OR “beta-Lactam” OR “beta Lactam” OR “beta Lactam Antibacterials” OR “Antibacterial Agents” OR “beta-Lactam Antibiotics” OR “beta Lactams” OR “Monocyclic” OR “beta-Lactams” OR “Monocyclic beta Lactam” OR “Monocyclic beta-Lactams” OR “Monocyclic beta Lactams” OR “Monocyclic beta-Lactam” OR “Amido-beta-Lactam Hydrolase” OR “Cephalosporin” OR “Cephalosporin Amido-beta-Lactam Hydrolase” OR “Cephalosporin Amido beta Lactam Hydrolase” OR “Hydrolase, Cephalosporin Amido-beta-Lactam”):ti,ab,kw  #7 “continuous” OR “extended” OR “prolonged” OR “intermittent” OR “interval” OR “discontinuous” OR “pulse” OR “bolus”  #8 MeSH descriptor: [Sepsis] explode all trees  #9 (“Pyemias” OR “Pyaemia” OR “Pyemia” OR “Pyohemias” OR “Pyohemia” OR “Pyaemias” OR “Blood Poisonings” OR “Blood Poisoning” OR “Poisoning, Blood” OR “Poisonings, Blood” OR “Septicemia” OR “Septicemias” OR “Bloodstream Infection” OR “Infection” OR “Bloodstream” OR “Bloodstream Infections” OR “Severe Sepsis” OR “Sepsis, Severe”):ti,ab,kw  #10 MeSH descriptor: [Shock, Septic] explode all trees  #11 (“Shock, Endotoxic” OR “Shocks, Endotoxin” OR “Endotoxin Shocks” OR “Shock, Endotoxin” OR “Endotoxin Shock” OR “Septic Shock” OR “Toxic Shock” OR “Toxic Shock Syndromes” OR “Shock, Toxic” OR “Toxic Shock Syndrome” OR “Shock Syndrome, Toxic”):ti,ab,kw  #12 MeSH descriptor: [Critical Care] explode all trees  #13 MeSH descriptor: [Critical Illness] explode all trees  #14 MeSH descriptor: [Intensive Care Units] explode all trees  #15 (“Critical Care” OR “Care, Critical” OR “Intensive Care” OR “Care, Intensive” OR “Surgical Intensive Care” OR “Care, Surgical Intensive” OR “Intensive Care, Surgical” OR “Critical Illness” OR “Critical Illnesses” OR “Illness, Critical” OR “Illnesses, Critical” OR “Critically Ill” OR “Intensive Care Units” OR “Intensive Care Unit” OR “Unit, Intensive Care” OR “ICU Intensive Care Units”):ti,ab,kw  #16 #1 OR #2 OR #3 OR #4 OR #5 OR #6  #17 #8 OR #9 OR #10 OR #11  #18 #12 OR #13 OR #14 OR #15  #19 #16 AND #7 AND #17 AND #18 | 366  11,509  1  226  2  31,752  272,440  6,463  111,921  1,401  3,909  3,079  3,653  6,002  38,134  36,949  116,762  38,422  379 |

Supplemental Table 3: Studies Meeting Inclusion Criteria but Excluded: Reasons for Exclusion.

| Studies Meeting Inclusion Criteria but Excluded，year | Reasons for Exclusion |
| --- | --- |
| Georges et al, 2005(1) | Other beta-lactam antibiotics were predominant |
| Rafati et al, 2006(2) | Other beta-lactam antibiotics were predominant |
| Lorente et al, 2006(3) | Only pharmacokinetic outcome |
| Roberts et al, 2007(4) | Other beta-lactam antibiotics were predominant |
| Roberts et al, 2009(5) | Only pharmacokinetic outcome |
| Roberts et al, 2010(5) | Other beta-lactam antibiotics were predominant |
| Dulhunty et al, 2013(6) | Other beta-lactam antibiotics were predominant |
| Dulhunty et al, 2015(7) | Other beta-lactam antibiotics were predominant |
| Jamal et al, 2015(8) | Only pharmacokinetic outcome |
| Jamal et al, 2015(9) | Only pharmacokinetic outcome |
| Abdul-Aziz et al, 2016(10) | Only pharmacokinetic outcome |
| Abdul-Aziz et al, 2016(11) | Other beta-lactam antibiotics were predominant |
| Taccone et al, 2016(12) | comments |
| Shabaan et al, 2017(13) | Only pharmacokinetic outcome |
| Yu et al, 2018(14) | Review |
| Mirjalili et al, 2023(15) | Other beta-lactam antibiotics were predominant |
| Álvarez-Moreno et al, 2024(16) | Other beta-lactam antibiotics were predominant |
| Ai et al, 2024(17) | Review |

Supplemental Table 4. Characteristics of the Included Participants in each trial

| Included trials | Continuous infusion | | | | | |  | Intermittent infusion | | | | | |
| --- | --- | --- | --- | --- | --- | --- | --- | --- | --- | --- | --- | --- | --- |
|  | Age ^b^ (years) | Male^a^ | APACHE II ^b^ | SOFA ^b^ | Leucocytes (x 10^9^/L) | CRP (mg/L) |  | Age ^b^ (years) | Male^a^ | APACHE II | SOFA | Leucocytes (x 10^9^/L) | CRP (mg/L) |
| Chytra et al.(18) 2012 | 44.9 ± 17.8 | 78 (65) | 21.4 ± 7.9 | 10.4 ± 2.9 | 15.9(11.5-21.1)^c^ | 155 (115-197) ^c^ |  | 47.2 ± 16.3 | 83 (69.2) | 22.1 ± 8.79 | 10.6 ± 3.5 | 14.1 (11.2-19.6) ^c^ | 199 (117 - 262) |
| Wang et al.(19) 2014 | 63.5±15.3 | 25(65.8) | 20.7±7.4  ^b^ | 6.0±2.5 | NR | NR |  | 57.2±19.5 | 34(85.0) | 19.2±7.0 | 6.8±2.6 | NR | NR |
| Helmy et al.(20)  2015 | NR | NR | NR | 9.94±0.93 | 25.28 ± 3.07 ^b^ | 228.18 ± 14.10 ^b^ |  | NR | NR | NR | 10.38±1.44 | 26.34 ± 2.75 ^b^ | 232.86 ± 10.20 |
| Zhao et al.(21) 2017 | 68.0 ± 15.4 | 10 (40.0) | 19.4 ± 5.0 | 8.0 ± 2.8 | 11.5 ± 4.0 ^b^ | 1.3 (0.3–4.0) ^e^ |  | 67.0 ± 12.2 | 11 (44.0) | 19.7 ± 5.9 | 8.5 ± 2.4 | 11.9 ± 5.0 ^b^ | 1.2 (0.3–23.8) ^e^ |
| Monti et al.(22) 2023 | 65.5 ± 14.0 | 195 (64) | 44 (35-55) ^d^ | 9 (6-11) ^c^ | NR | NR |  | 63.4 ± 15.0 | 209 (69) | 43 (34-53) ^d^ | 9 (6-11) ^c^ | NR | NR |

^a^ Data are number of patients (percentage); ^b^ Mean ± SD; ^c^ Median [interquartile range] ; ^d^ Simplified Acute Physiology Score II; ^e^ Procalcitonin(μg/L).

Abbreviation: NR, not reported; APACHE, acute physiology and chronic health evaluation; SOFA, sequential organ failure assessment

Supplemental Table 5. Type of infection and the proportion of patients

| Included trials | Abdominal, n  (%) CI vs II | Respiratory, n  (%) CI vs II | Bloodstream, n  (%) CI vs II | Uroinfection, n  (%) CI vs II | others, n  (%) CI vs II |
| --- | --- | --- | --- | --- | --- |
| Chytra et al.(18) 2012 | 23 (19.2%) vs  31 (25.8%) | 66 (55.0%) vs  61 (50.8%) | 10 (8.3%) vs  11 (9.2%) | 11 (9.2%) vs  6 (5.0%) | 7 (5.8%) vs  11 (9.2%) |
| Wang et al.(19) 2014 | NR | 38 (100.0%) vs  40 (100.0%) | NR | NR | NR |
| Helmy et al.(20)  2015 | NR | NR | NR | NR | NR |
| Zhao et al.(21) 2017 | 14 (56.0) vs  13 (52.0) | 9 (36.0) vs  10 (40.0) | 5 (20.0) vs  3 (12.0) | 1 (4.0) vs  2 (8.0) | 1 (4.0) vs  0 (0) |
| Monti et al.(22) 2023 | 28 (9.6) vs  24 ((21)8.1) | 96 (33) vs  99 (33) | 28 (9.6) vs  15 (5.1) | 16 (5.5) vs  16 (5.5) | 33 (11) vs  35 (12) |

Abbreviation: CI, Continuous infusion; II, Intermittent infusion; NR, not reported.

Supplemental Table 6. Summary of results of trial sequential analyses.

|  | Effect size | Incidence, or variance | I2  (%) | D2  (%) | RIS | % of RIS attained | Z-curve passed the conventional boundaries? | Z-curve passed the TSA boundaries? | Z-curve passed the futility boundaries? |
| --- | --- | --- | --- | --- | --- | --- | --- | --- | --- |
| All-cause mortality (5 studies, n = 1075) | RRR: 15.0% | 41.8% | 30 | 63 | 6823 | 15.8 | No ^a^ | No | No |
| ICU length of stay (5 studies, n = 1075) | MID 1 day | 26.8 | 40 | 76 | 4678 | 23.0 | Yes | No | No |
| Clinical cure rate (4 studies, n = 442) | RRR: 15.0% | 75% | 20 | 40 | 1170 | 37.8 | Yes | No | No |
| Duration of meropenem therapy (3 studies, n = 368) | MID 1 day | 4.8 | 20 | 40 | 749 | 49.1 | Yes | No | No |

Abbreviation: ICU, Intensive care unit, D^2^: diversity, I^2^: inconsistency, MID: minimally important difference, RIS: required information size, RD: risk difference, RRR: relative risk reduction.
^a^ The initial observation indicates a statistically significant effect, but as additional data or factors are considered, the effect diminishes over time and eventually becomes non-significant

**
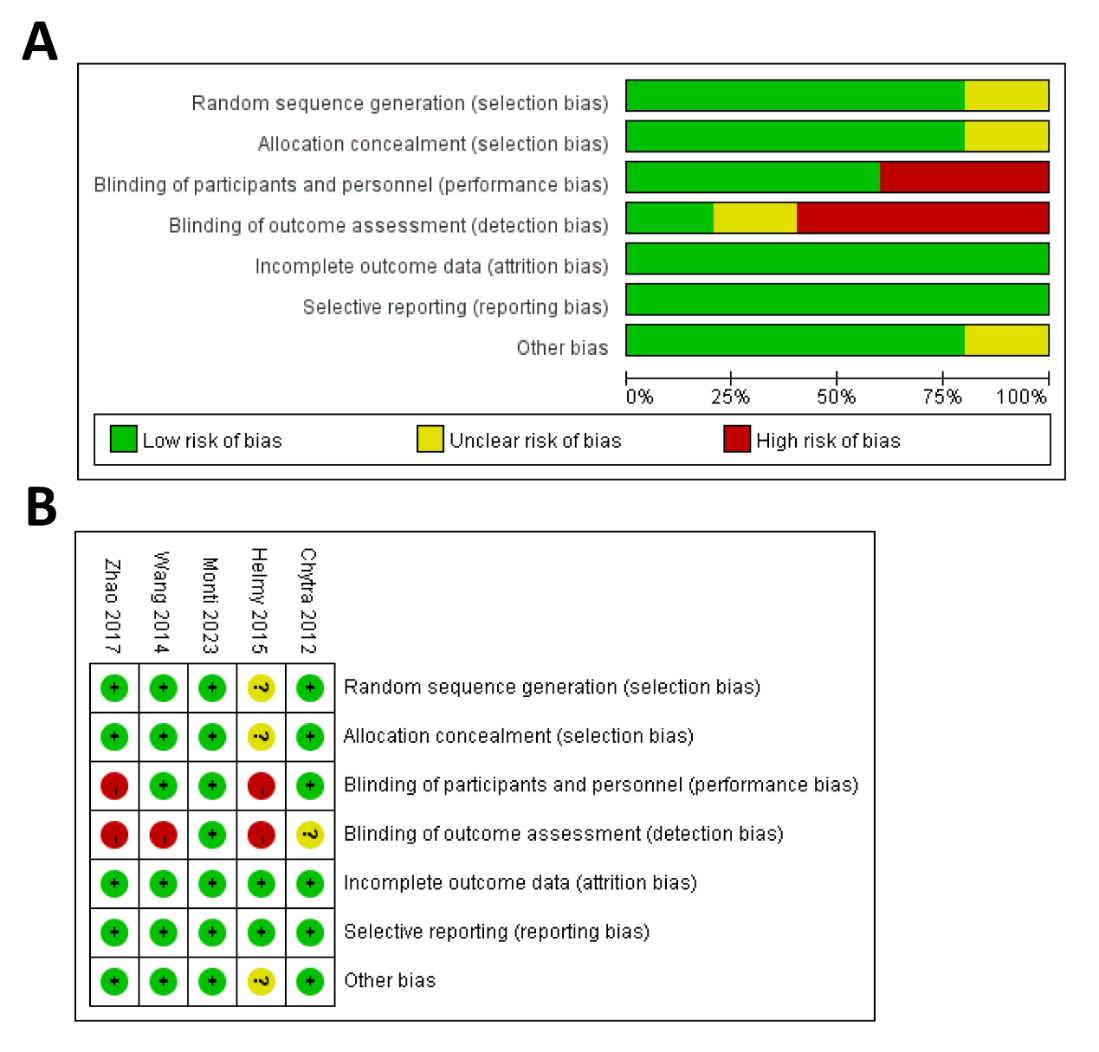
**

Supplemental Figure 1. Risk of bias graph and risk of bias summary graph. Risk of bias graph: review authors’ judgements about each risk of bias item presented as percentages across all included studies (A). Risk of bias summary graph: review authors’ judgements about each risk of bias item for each included study (B).


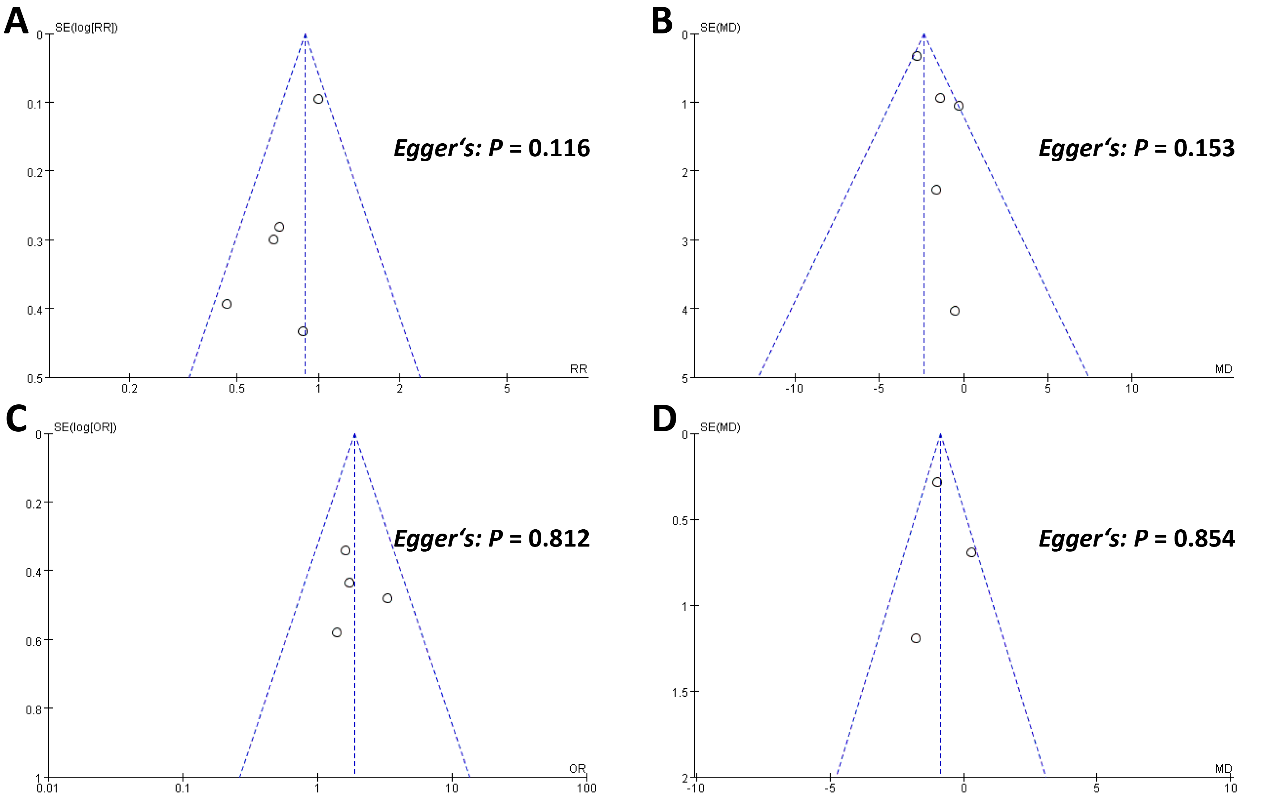


Supplemental Figure 2. Funnel plots. All-cause mortality (A), ICU length of stay (B), clinical cure rate (C) and duration of meropenem therapy (D).


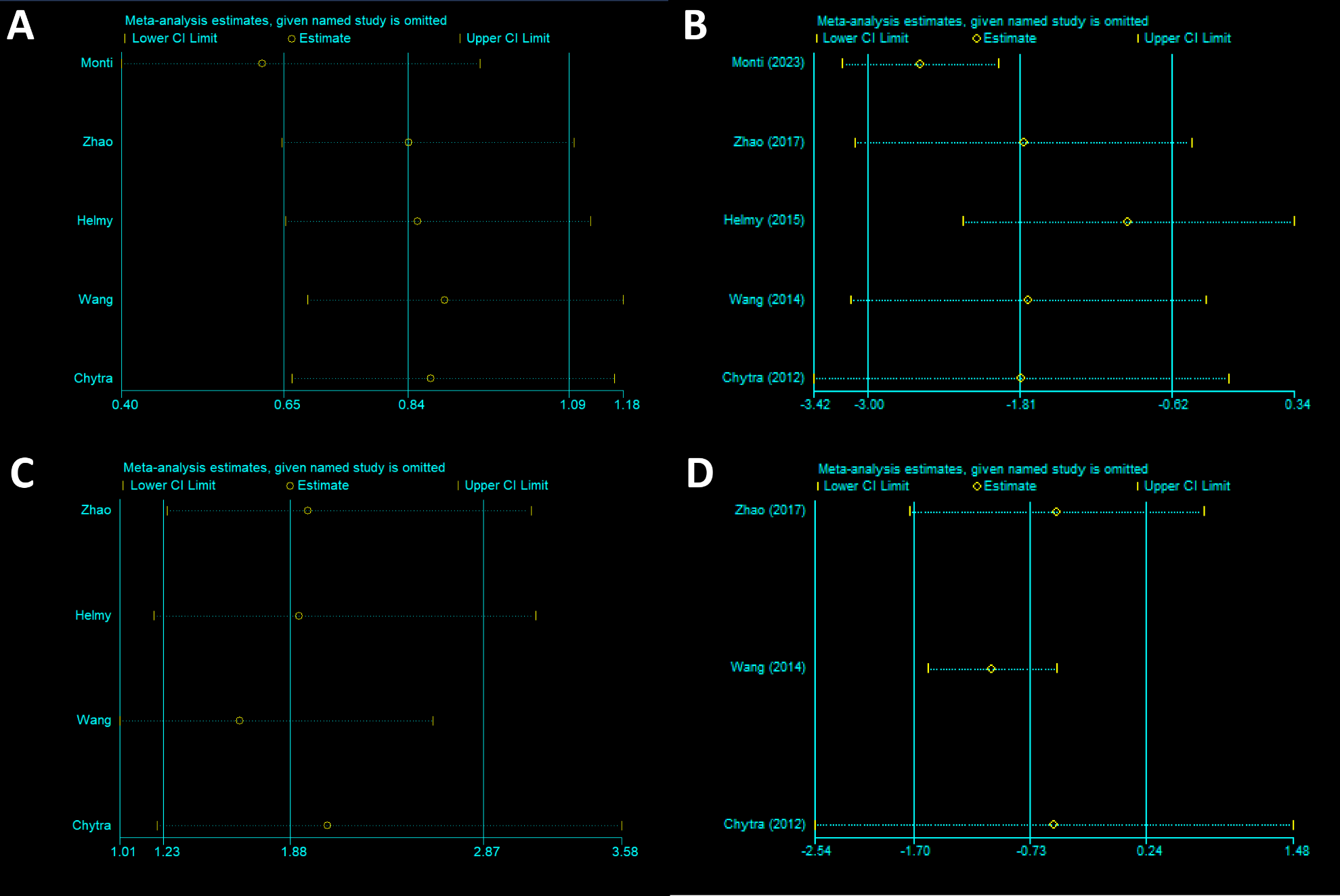
 Supplemental Figure 3. Sensitivity analyses clinical and nutritional outcomes. All-cause mortality (A), ICU length of stay (B), clinical cure rate (C) and duration of meropenem therapy (D).

# References

1. Georges B, Conil JM, Cougot P, Decun JF, Archambaud M, Seguin T, et al. Cefepime in critically ill patients: continuous infusion vs. an intermittent dosing regimen. Int J Clin Pharmacol Ther. 2005;43(8):360-9.

2. Rafati MR, Rouini MR, Mojtahedzadeh M, Najafi A, Tavakoli H, Gholami K, et al. Clinical efficacy of continuous infusion of piperacillin compared with intermittent dosing in septic critically ill patients. Int J Antimicrob Agents. 2006;28(2):122-7.

3. Lorente L, Lorenzo L, Martín MM, Jiménez A, Mora ML. Meropenem by continuous versus intermittent infusion in ventilator-associated pneumonia due to gram-negative bacilli. Ann Pharmacother. 2006;40(2):219-23.

4. Roberts JA, Boots R, Rickard CM, Thomas P, Quinn J, Roberts DM, et al. Is continuous infusion ceftriaxone better than once-a-day dosing in intensive care? A randomized controlled pilot study. J Antimicrob Chemother. 2007;59(2):285-91.

5. Roberts JA, Kirkpatrick CM, Roberts MS, Robertson TA, Dalley AJ, Lipman J. Meropenem dosing in critically ill patients with sepsis and without renal dysfunction: intermittent bolus versus continuous administration? Monte Carlo dosing simulations and subcutaneous tissue distribution. J Antimicrob Chemother. 2009;64(1):142-50.

6. Dulhunty JM, Roberts JA, Davis JS, Webb SA, Bellomo R, Gomersall C, et al. Continuous infusion of beta-lactam antibiotics in severe sepsis: a multicenter double-blind, randomized controlled trial. Clin Infect Dis. 2013;56(2):236-44.

7. Dulhunty JM, Roberts JA, Davis JS, Webb SA, Bellomo R, Gomersall C, et al. A Multicenter Randomized Trial of Continuous versus Intermittent β-Lactam Infusion in Severe Sepsis. Am J Respir Crit Care Med. 2015;192(11):1298-305.

8. Jamal JA, Mat-Nor MB, Mohamad-Nor FS, Udy AA, Wallis SC, Lipman J, et al. Pharmacokinetics of meropenem in critically ill patients receiving continuous venovenous haemofiltration: a randomised controlled trial of continuous infusion versus intermittent bolus administration. Int J Antimicrob Agents. 2015;45(1):41-5.

9. Jamal JA, Roberts DM, Udy AA, Mat-Nor MB, Mohamad-Nor FS, Wallis SC, et al. Pharmacokinetics of piperacillin in critically ill patients receiving continuous venovenous haemofiltration: A randomised controlled trial of continuous infusion versus intermittent bolus administration. Int J Antimicrob Agents. 2015;46(1):39-44.

10. Abdul-Aziz MH, Lipman J, Akova M, Bassetti M, De Waele JJ, Dimopoulos G, et al. Is prolonged infusion of piperacillin/tazobactam and meropenem in critically ill patients associated with improved pharmacokinetic/pharmacodynamic and patient outcomes? An observation from the Defining Antibiotic Levels in Intensive care unit patients (DALI) cohort. J Antimicrob Chemother. 2016;71(1):196-207.

11. Abdul-Aziz MH, Sulaiman H, Mat-Nor MB, Rai V, Wong KK, Hasan MS, et al. Beta-Lactam Infusion in Severe Sepsis (BLISS): a prospective, two-centre, open-labelled randomised controlled trial of continuous versus intermittent beta-lactam infusion in critically ill patients with severe sepsis. Intensive Care Med. 2016;42(10):1535-45.

12. Taccone FS, Laupland KB, Montravers P. Continuous infusion of β-lactam antibiotics for all critically ill patients? Intensive Care Med. 2016;42(10):1604-6.

13. Shabaan AE, Nour I, Elsayed Eldegla H, Nasef N, Shouman B, Abdel-Hady H. Conventional Versus Prolonged Infusion of Meropenem in Neonates With Gram-negative Late-onset Sepsis: A Randomized Controlled Trial. Pediatr Infect Dis J. 2017;36(4):358-63.

14. Yu Z, Pang X, Wu X, Shan C, Jiang S. Clinical outcomes of prolonged infusion (extended infusion or continuous infusion) versus intermittent bolus of meropenem in severe infection: A meta-analysis. PLoS One. 2018;13(7):e0201667.

15. Mirjalili M, Zand F, Karimzadeh I, Masjedi M, Sabetian G, Mirzaei E, et al. The clinical and paraclinical effectiveness of four-hour infusion vs. half-hour infusion of high-dose ampicillin-sulbactam in treatment of critically ill patients with sepsis or septic shock: An assessor-blinded randomized clinical trial. J Crit Care. 2023;73:154170.

16. Álvarez-Moreno CA, Nocua-Báez LC, Ortiz G, Torres JC, Montenegro G, Cervera W, et al. Efficacy of Continuous vs. Intermittent Administration of Cefepime in Adult ICU Patients with Gram-Negative Bacilli Bacteremia: A Randomized Double-Blind Clinical Study. Antibiotics (Basel). 2024;13(3).

17. Ai MY, Chang WL, Liu CY. Mortality of continuous infusion versus intermittent bolus of meropenem: a systematic review and meta-analysis of randomized controlled trials. Front Microbiol. 2024;15:1337570.

18. Chytra I, Stepan M, Benes J, Pelnar P, Zidkova A, Bergerova T, et al. Clinical and microbiological efficacy of continuous versus intermittent application of meropenem in critically ill patients: a randomized open-label controlled trial. Crit Care. 2012;16(3):R113.

19. Wang Z, Shan T, Liu Y, Ding S, Li C, Zhai Q, et al. [Comparison of 3-hour and 30-minute infusion regimens for meropenem in patients with hospital acquired pneumonia in intensive care unit: a randomized controlled clinical trial]. Zhonghua Wei Zhong Bing Ji Jiu Yi Xue. 2014;26(9):644-9.

20. Mohamed ESW, Pharm J, Sci B, Abdallah T, Helmy A, Ahmed abdelghaffar EM, et al., editors. CONTINUOUS VERSUS INTERMITTENT INTRAVENOUS MEROPENEM IN SEVERE SEPSIS2015.

21. Zhao HY, Gu J, Lyu J, Liu D, Wang YT, Liu F, et al. Pharmacokinetic and Pharmacodynamic Efficacies of Continuous versus Intermittent Administration of Meropenem in Patients with Severe Sepsis and Septic Shock: A Prospective Randomized Pilot Study. Chin Med J (Engl). 2017;130(10):1139-45.

22. Monti G, Bradic N, Marzaroli M, Konkayev A, Fominskiy E, Kotani Y, et al. Continuous vs Intermittent Meropenem Administration in Critically Ill Patients With Sepsis: The MERCY Randomized Clinical Trial. Jama. 2023;330(2):141-51.
